# Reduced categorical learning of faces in dyslexia

**DOI:** 10.1016/j.cortex.2024.01.005

**Published:** 2024-04

**Authors:** Ayelet Gertsovski, Odeya Guri, Merav Ahissar

**Affiliations:** a The Edmond and Lily Safra Center for Brain Sciences, The Hebrew University of Jerusalem, Jerusalem, Israel; b Department of Cognitive and Brain Sciences, The Hebrew University of Jerusalem, Jerusalem, Israel

**Keywords:** Perceptual learning, Dyslexia, Drift diffusion model, Face categorization

## Abstract

The perception of phonological categories in dyslexia is less refined than in typically developing (TD) individuals. Traditionally, this characteristic was considered unique to phonology, yet many studies showed non-phonological perceptual difficulties. Importantly, measuring the dynamics of cortical adaptation, associated with category acquisition, revealed a broadly distributed faster decay of cortical adaptation. Taken together, these observations suggest that the acquisition of perceptual categories in dyslexia may be slower across modalities. To test this, we tested adult individuals with developmental dyslexia (IDDs) and TDs on learning of two unknown faces, yielding face-specific categorization. Initial accuracy was similar in the two groups, yet practice-induced increase in accuracy was significantly larger in TDs. Modeling the learning process (using Drift Diffusion Model) revealed that TDs' steeper learning results from a larger increase in their effective face-specific signal. We propose that IDDs' slower item-specific categorical learning of unknown faces indicates that slower categorical learning in dyslexia is a core, domain-general difficulty.

## 1 Introduction

Developmental dyslexia is a specific impairment in acquiring reading skills that is not accounted for by age, vision problems or inadequate schooling (World Health Organization, 2008). Individuals with developmental dyslexia (IDDs) commonly have difficulties in additional language-associated tasks, mainly phonological awareness and verbal short-term memory (Vellutino et al., 2004). Prominent theories suggest that IDDs have a deficit in phonological representations (e.g., Snowling, 2000) or in accessing these representations (Ramus, 2014; Ramus & Szenkovits, 2008). Indeed, categorical perception of speech sounds was found to be reduced in IDDs compared with typically developing (TD) individuals (e.g., Noordenbos & Serniclaes, 2015; Werker & Tees, 1987), as was implicit learning of novel speech sound categories (Vandermosten et al., 2019).

However, IDDs also have difficulties in non-linguistic perceptual tasks (e.g., Ahissar et al., 2000; Ramus et al., 2003; Sperling et al., 2005). The ‘anchoring deficit’ hypothesis (Ahissar, 2007; Ahissar et al., 2006) accounts for these difficulties by suggesting that IDDs' item-specific learning is reduced due to a faster decay of their implicit memory trace (Jaffe-Dax et al., 2017; Lieder et al., 2019). Imaging studies using auditory stimuli associated this faster decay with IDDs' reduced adaptation to stimulus regularities (Gertsovski & Ahissar, 2022; Jaffe-Dax et al., 2017). Importantly, shorter cortical adaptation in dyslexia was found for a broad range of stimuli, including visual objects and faces (Perrachione et al., 2016), and in broadly distributed cortical regions (Jaffe-Dax et al., 2018), suggesting a domain-general property. These findings suggest that category learning in dyslexia might be slower in general, since a shallower exposure-based learning slope is expected to reduce the rate of category formation and enrichment (Banai & Ahissar, 2018; Kimel et al., 2020). To test this hypothesis, we studied category learning of faces, as a specific case study for a potentially general deficit.

Faces are very different from stimuli with which IDDs are traditionally considered to have difficulties. There is evidence for a right-hemisphere dominance of face processing (e.g., Kanwisher et al., 1997), as opposed to the left-hemisphere dominance of language and written words (McCandliss et al., 2003). Yet, like words, expertise for specific faces is gained and developed during years of exposure (reviewed in Young & Burton, 2018). Thus, if IDDs' reduced adaptation duration is associated with reduced categorical learning – and given the evidence that it is a general property, not specific to adaptation of auditory or linguistic stimuli, and was specifically found for faces – we expect to see a shallower learning slope also for face categories.

Face processing was previously studied in dyslexia, with mixed findings. Several studies found reduced face recognition in IDDs (e.g., Collins et al., 2017; Gabay et al., 2017; Sigurdardottir et al., 2015, 2018, 2019), while others did not (e.g., Kühn et al., 2021; Rüsseler et al., 2003). Interestingly, some of these results support an expertise-based account of IDDs’ difficulties in face recognition. For example, Sigurdardottir et al. (2018) found poorer performance of IDDs in matching faces, but not in an identical task with novel objects and scrambled faces – stimuli with which the participants had no expertise. In addition, Smith-Spark and Moore (2009) did not find a general group difference in naming famous faces, but they did find that TDs (and not IDDs) responded faster to faces that were acquired earlier, suggesting that IDDs are less sensitive to the amount of experience with these faces. Still, none of these studies tracked the process of learning specific faces in dyslexia. Thus, the question of whether there is a shallower learning slope for face categories in dyslexia remains open.

We now tracked the initial stages of face-specific category learning. We administered a categorization task with morphed unfamiliar faces to adult TDs and IDDs, and tested learning of face categorization across two sessions. To ensure that sluggish attention (Hari & Renvall, 2001) does not underlie potential group differences, we trained (and assessed performance) under several inter-trial intervals (2, 3.5 and 5 sec). This allowed us to assess whether relaxing the attentional load by elongating the intervals between adjacent stimuli yields a larger benefit to IDDs. Finally, we modeled our results using the drift diffusion model (DDM; Ratcliff, 1978; Ratcliff & McKoon, 2008), a decision making model which integrates RT and accuracy into a unified conceptual framework. Using the model, we were able to separate the decision making process into different parameters, and ask whether group differences in category learning are related to changes in the efficiency of information accumulation during the task, to changes in strategies of decision making, or to non-decisional processes.

## 2 Methods

### 2.1 Participants

Participants were recruited using ads posted at the Hebrew University of Jerusalem and other colleges in Jerusalem, Israel. All participants completed a demographic questionnaire, including previous diagnoses of learning disorders and relevant medical conditions. Based on this questionnaire, we invited adult native Hebrew speakers who either reported having no reading or learning difficulties, or had a diagnosis of a specific reading disorder by an authorized clinician. All invited participants reported that they did not have an uncorrected vision problem, and that they did not take psychiatric medications other than for attention deficit. All participants gave their informed consent to take part in this study and were paid for their participation. This study was approved by the Ethics Committee of the Psychology Department of the Hebrew University.

Participants were first administered a screening session (for assessing general cognitive and reading skills, described below). Participants from both groups were invited to participate in the face categorization task (described below) only if their scaled score in the Block Design, a non-verbal visuo-spatial reasoning task (the Hebrew version of WAIS-III; Wechsler, 1997) was at least 7 (i.e., equal to or higher than 1 SD below the population mean, 10). This criterion was established prior to data analysis.

Fifty-six participants, 27 IDDs and 29 TDs, completed the face categorization task. All participants, except for 2 IDDS and 2 TDs, either had a university or college degree, or were in the process of studying for it. 11 of the IDDs reported that they are taking medication for attention deficit (they were asked not to take it before their participation in experimental sessions). There was no difference in category learning between the two IDD subgroups based on this self-report. We therefore treated IDD participants as a single group. All participants performed two sessions, each lasting ∼35 min, with a ∼20 min break between them. Learning was calculated as the difference in performance between the two sessions. The sample size was determined based on training studies of TDs (training session conducted in a single day without intervening sleep periods), which reported an effect size of d_z_ > .6 (e.g., Atienza et al., 2002; Banai & Lavner, 2014; Hervais-Adelman et al., 2008). To detect such an effect with 80% power, at least 24 participants are required. We assumed that a similar number of IDDs will be sufficient to detect a similar learning effect in the dyslexia group, if it exists.

Two participants were excluded based on their performance in the face categorization task: One TD, who had 5 blocks with more than 20% trials with no recorded response, and one IDD whose performance in the second session was at chance level. The results are thus reported for 26 IDDs (16 females) and 28 TDs (10 females).

### 2.2 Cognitive assessments in the screening session

1. Non-verbal reasoning was measured with the standard Block Design visuo-spatial reasoning task (the Hebrew version of WAIS-III; Wechsler, 1997).
2. Short-term verbal memory was measured using the standard Digit Span task, consisting of the Digit Forward and Backward subtests (the Hebrew version of WAIS-III; Wechsler, 1997).
3. Phonological decoding was assessed using a list of 24 pseudo-words in Hebrew characters with diacritics (Deutsch & Bentin, 1996). Participants were instructed to read the words aloud, as quickly and accurately as possible.
4. Reading fluency was assessed by reading a four-paragraph academic-level text (adapted from Ben-Yehudah et al., 2001). Participants were instructed to read the text aloud, as quickly and accurately as possible, but slow enough to be able to answer a simple content question at the end.
5. Phonological awareness was assessed using the spoonerism task, in which participants heard 20 two-word expressions in Hebrew, and were asked to swap the initial phonemes of the two words and respond vocally (Ben-Yehudah & Ahissar, 2004; Ben-Yehudah et al., 2001).
6. Spelling skills were measured with a task in which participants were presented with 26 written word pairs, each composed of a real word and a homophone that contained a spelling error (Ben-Yehudah et al., 2001). Participants were requested to mark the correctly spelled word as quickly as possible.

In all the phonological and reading tasks, both accuracy and rate were scored. As expected, TDs scored significantly higher than IDDs in all the reading, spelling and phonological tasks, and in the Digit Span task. Lower scores in the Digit Span task have been consistently reported in dyslexia (e.g., Gallagher et al., 2000; Helland & Asbjørnsen, 2004). They are typically interpreted as reflecting poor phonological memory. Importantly, there was no significant group difference in age or in non-verbal reasoning skills (Table 1).

**Table 1:** Mean, SD and Cohen's d for scores in cognitive assessments.

Assessment	TDs (N = 28) Mean (SD)	IDDs (N = 26) Mean (SD)	Group difference Cohen's d
Age (years)	25.6 (4.6)	27.2 (4.9)	−.35, ns
General cognitive tests (scaled WAIS score)			
Block design	12.9 (2.6)	12.3 (2.6)	.21, ns
Digit span	11.6 (2.5)	8 (2.6)	1.42∗∗∗
Reading rate (items/minute)			
Pseudo-word reading rate	62.5 (25.5)	31.9 (10.2)	1.56∗∗∗
Paragraph reading rate	134.2 (24.8)	90.5 (23)	1.83∗∗∗
Reading accuracy (% correct)			
Pseudo-word reading accuracy	85.1 (16.3)	61.2 (15.9)	1.49∗∗∗
Paragraph reading accuracy	97.9 (1.4)	93.8 (3)	1.76∗∗∗
Phonological awareness			
Spoonerism rate (items/minute)	11.6 (4)	4.7 (1.7)	2.23∗∗∗
Spoonerism accuracy (% correct)	91.3 (9.1)	71.5 (21.1)	1.24∗∗∗
Spelling			
Spelling rate (items/minute)	75.3 (13)	43.2 (20.4)	1.9∗∗∗
Spelling accuracy (% correct)	99.3 (1.8)	93.1 (11.8)	.76∗∗
Summary z-score	0 (.7)	−2.2 (1.5)	1.9∗∗∗

The summary z-score is the mean z-score of accuracy and rate of pseudo-word and paragraph reading, spelling, and spoonerism (all measures were normalized based on TD performance). WAIS = Wechsler Adult Intelligence Scale; ns = not statistically significant (*p* < .05), ∗∗*p* < .01, ∗∗∗*p* < .001 in an independent t-test between the groups.

### 2.3 Stimuli

**Fig. 1:**
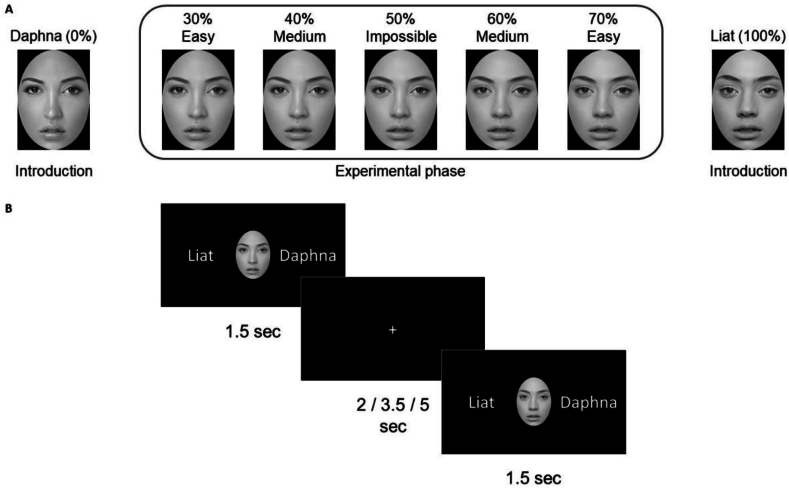
Stimuli and procedure. (A) The seven stimuli used in the experiment: Two endpoint faces of “Daphna” and “Liat”, marked as 0% and 100% according to the percent of the Liat face in them, were used in the introduction phase. Five morphed faces, marked as 30%, 40%, 50%, 60% and 70%, were used in the experimental phase. (B) Two example test trials. In each trial, participants were shown a morphed face for 1.5 sec, and were asked to determine whether the face was of Liat or of Daphna. These names were shown alongside the face (written in Hebrew in the actual experiment). Between the trials, a fixation cross was shown. The inter-trial interval (ITI) was either 2, 3.5 or 5 sec, in separate blocks.

The stimuli were created based on two images of unfamiliar female faces taken from the internet. The images were converted to grayscale and were edited to make them similar in brightness and in size. An oval region of the face was cropped, leaving the hair and ears out. The resulting image resolution was 284 × 395 pixels. The two faces were labeled with Israeli names: “Daphna” and “Liat” (Fig. 1A), which are both 4-letter words in Hebrew writing. The two faces were morphed (using the Sqirlz Morph software, Xiberpix, version 2.1), resulting in a continuum of 11 morphed images with a 10% interval between them, starting with the endpoint “Daphna” face, to 10% (i.e., 10% Liat and 90% Daphna), 20%, 30%, 40% Liat and so forth, up to the endpoint 100% Liat face. The two endpoint faces were used in the introduction phase. In the main experimental phase, we chose to use a narrow subsection of the continuum in order to make the task more difficult, hence only the 30%, 40%, 50%, 60% and 70% Liat faces were used (Fig. 1A). The faces were presented on a black background.

### 2.4 Experimental procedure

The experiment was programmed using Matlab with the Psychtoolbox extension (Psychophysics Toolbox version 3.0.16, Brainard, 1997), and was presented on a 24-inch monitor (1980 × 1020 pixels). Participants were seated 70 cm from the monitor. At the beginning of each session, participants were familiarized with the two endpoint faces. Each face was presented once on the screen alongside its labeled name (Daphna or Liat) for 15 sec. The participants were asked to memorize the face of each woman and her name.

The experiment was composed of a short introductory session, followed by two experimental sessions. Both introduction and the experimental sessions had the same basic trial structure: in each trial, a single face was presented in the middle of the screen, and its two potential categories (names) were presented on the two sides (Fig. 1B). The participants were asked to categorize the face as Daphna's or Liat's, by pressing the designated keyboard key whose position matched the position (left/right) of the category on the screen. The locations of the categories were counterbalanced between participants, and were constant during the experiment. The face was presented for 1.5 sec, during which the participants were allowed to respond. The face remained on screen during this time regardless of the participant's response. If no response was recorded during this time period, a “No response was detected!” message appeared for 1 sec, and the trial was later removed from the analyses. Between trials, a white fixation cross appeared in the middle of the screen on a black background. The duration of the fixation cross, i.e., the inter-trial interval (ITI), was manipulated as a condition of interest in the experiment, as detailed below.

The introduction phase included only the two endpoint faces, and feedback was presented visually for 1 sec after each trial. The ITI was fixed at 2 sec. Each face was presented 10 times, at a random order. If the introduction accuracy was below 85%, the introduction session was administered again. One TD and one IDD completed the introduction session 3 times before reaching this threshold. All other participants needed a single session or two. IDDs needed marginally more introduction sessions to pass the threshold (mean ± SD = 1.46 ± .58) compared with TDs [mean ± SD = 1.18 ± .48; t(52) = −1.96, *p* = .055].

In the experimental phase, only the 5 morphed faces were used (30%, 40%, 50%, 60% and 70% Liat). Each stimulus appeared 139–148 times, and the order was randomized. No accuracy feedback was provided at this phase. The participants were given the same basic instructions as in the introduction, but they were also told that at this stage it may be more difficult to recognize the faces. In addition, they were told that the two women will appear a similar number of times, to encourage them to distinguish between the two face identities. The experimental phase was composed of two equally-structured sessions. Between these two sessions, the participants were asked to take a break of about 20 min, and were offered refreshments. To test the effect of attentional load on task performance and learning, we manipulated the ITI, since longer ITIs reduce attentional load (e.g., as manifested in attentional blink, Shapiro et al., 1997). Specifically, we wanted to test whether elongating the ITI has a larger effect on IDDs given the findings that they might have a longer attentional blink window in dyslexia (Hari et al., 1999, though see Badcock et al., 2008). There were three ITI conditions, in which the duration of the interval between trials (in which a fixation cross was shown) was either 2, 3.5 or 5 sec. The experimental phase was divided to blocks of 60 trials each, and the ITI of each block was fixed. The ITI of the first block was counterbalanced between participants, and there were no consecutive blocks with the same ITI. Each experimental session included two blocks of each ITI, which amounted to 360 trials per session. The participants were invited to take a short break between blocks. In most cases, the participants chose to continue to the next block in less than a minute, but occasionally they took a few minutes' break.

### 2.5 Behavioral data analysis

In each trial, participants were required to respond within 1.5 sec of stimulus presentation. Trials in which no response was recorded during this time period were removed from further analyses. Blocks with more than 20% no-response trials were removed. In addition to the complete exclusion of one TD participant due to a large number of such blocks (see Participants above), this analysis affected the data of two IDDs, who each had a single block removed. The mean percent of remaining no-response trials was small in both groups, yet was smaller in the TD (mean ± SD = 2.2 ± 1.4%) than in the IDD group [mean ± SD = 3.22 ± 1.62%; t(52) = −2.47, *p* = .017].

To measure the discrimination sensitivity, we calculated d′ = z(hit rate) − z(false-alarm rate). Thus, d′ = 0 reflects chance level performance. For this analysis, we used the 30% and 40% Liat stimuli, which were considered a hit if the participant responded “Daphna”, and the 60% and 70% Liat stimuli, for which responses were defined as a hit if the participant responded “Liat”. Responses to the 50% stimulus were not included in the d′ analysis (since there was no correct answer). To allow for the estimation of d′ also in cases of extreme hit or false alarm rates, we applied a log-linear correction (Hautus, 1995). Reaction time (RT) analysis was based on all trials in which there was a response, including the 50% stimulus trials.

We used the 50% stimulus to calculate the response bias of each participant in each session. For 50% stimuli, which have no correct answer, this measure is orthogonal to signal enhancement (which we derived from drift diffusion modeling, explained below), and indicates how consistent a participant is with respect to her previous decisions for the same stimulus. We calculated response bias as the absolute value of the distance between the mean percent of “Liat” responses to that stimulus and 50% (chance level performance) in each experimental session.

### 2.6 Drift diffusion model (DDM)

**Fig. 2:**
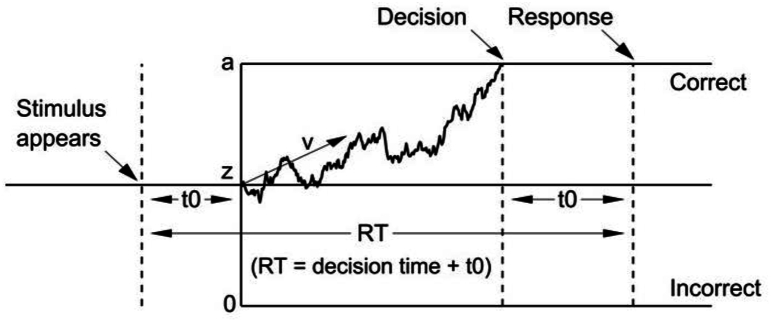
An illustration of the DDM. A simulated path depicts the noisy accumulation of evidence over time, with a drift rate v, which denotes the rate of information accumulation. The upper boundary denotes the threshold for a correct response (e.g., “Liat” for the 60% Liat morph), and the lower boundary denotes the threshold for an incorrect response. Accuracy is based on the face most similar to the stimulus, hence trials with a 50% stimulus, for which there was no correct response, were not included in the DDM modeling. The distance between the two boundaries is denoted by the threshold separation parameter – a. The starting point of the evidence accumulation path is denoted by z, and in our implementation it was fixed at a/2 (no bias). The non-decision time t0 is a single parameter which represents the duration of all non-decisional processes, both before and after the evidence accumulation process. RT is the sum of the decision time and t0.

We modeled our results using the DDM (Ratcliff, 1978; Ratcliff & McKoon, 2008). In this model, illustrated in Fig. 2, the decision process is modeled as a noisy process of evidence accumulation (enhancing the signal) over time via sequential sampling, until one of two boundaries is reached, and then a decision is made and a response is initiated. We used the DDM to ask which of three main model parameters explain the effects of session and of ITI on choices and RT in each of the groups: 1) The drift rate (v), which is the rate of information accumulation, and is larger when the signal is larger; 2) The non-decision time (t0), which is the mean duration of all non-decisional processes, including encoding and response execution; 3) The threshold separation (a), which is the distance between the two boundaries (related to the two potential decisions). The larger it is, the higher the participant sets the threshold of accuracy for making a decision.

We used the fast-dm software (version 30.2) (Voss et al., 2015) to fit the DDM to each participant's choices and RTs. Trials with RT < 200 msec were defined as fast outliers (Voss et al., 2013) and were removed before modeling. These trials were .02% and .12% of the trials in the TD and the IDD groups, respectively. On average, this means less than a single trial per participant. We estimated the models using the Maximum Likelihood method. To reduce the number of parameters, we coded choices in terms of accuracy, rather than by stimulus. That is, instead of coding the participant's response (“Liat” or “Daphna”), we coded whether they answered correctly or not. This allowed us to group the stimuli based on the difficulty level (easy: 30% and 70%, medium: 40% and 60%) instead of modeling each morph separately. Trials with the 50% stimulus, which have no accuracy value, were thus not included in the DDM analysis. The average number of trials (±STD) in each combination of difficulty level, ITI and session for each subject was 47.1 (±1.4) in the TD group and 46.2 (±3.2) in the dyslexia group. In an initial model, we allowed the drift rate (v) to vary with difficulty, session and ITI. Non-decision time (t0) and threshold separation (a) were both allowed to vary only with session and with ITI, because the participants had no information about the upcoming difficulty of the stimuli (which were interleaved), and hence we did not expect it to affect these parameters (Voss et al., 2013). In addition, the inter-trial variability of the non-decision time (st0) was free, as advised in order to improve model fitting (Voss et al., 2015). All other parameters were fixed at 0, except for the relative starting point (z_r_ = z/a), which was fixed at .5 (no bias). Since we coded responses based on accuracy, no bias was expected a-priory for or against the correct response. In a second model, to further reduce the number of parameters, we did not allow the non-decision time and the threshold separation to change with session. A model comparison using the Bayesian Information Criterion (BIC), which penalizes for the number of parameters, preferred the second, simpler model (mean BIC: 289.65) over the more complex first model (mean BIC: 294.52). Hence, the simpler model was used for all further analyses. Prior to statistical analysis, the estimated drift rates were averaged across difficulty levels.

We assessed model fit using a simulation analysis based on the estimated parameter values. For each of the two models, 1000 random parameter sets were drawn from a multidimensional normal distribution defined by the covariance matrix of the estimated parameter values. For each of these parameter sets, a single dataset was simulated using the construct-samples tool of fast-dm (Voss et al., 2015), with the same number of trials as the experimental dataset. The simulated datasets were then entered into the model with the same settings that were used to model the real data. To assess model fit, the 5% percentile of the distribution of the simulation-based fit indices (minus log likelihood) was taken as a critical value. For each of the two models, no real dataset had a worse fit than the critical value.

### 2.7 Statistical analysis

All statistical analyses were performed using R version 4.0.2 (R Core Team, 2020). We used mixed-design analyses of variance (ANOVAs) for all main analyses, using the afex package (Singmann et al., 2020). A separate ANOVA was run for each dependent variable: d′, RT, response bias (see definition above) and the DDM parameters drift rate (v), non-decision time (t0) and threshold separation (a). These ANOVAs included group (TD or IDD) as a between-subject variable and session (first or second) and ITI (2, 3.5 or 5 sec) as within-subject variables. Exceptions were 1) the analyses of the non-decision time and threshold separation parameters of the DDM, which did not include the session parameter, since the final model allowed them to vary only with ITI, and 2) the response bias analysis, which did not include the ITI parameter, since we were only interested in the change in bias by session. As a default in afex, the Greenhouse-Geisser correction was used in all ANOVAs. Post-hoc tests were conducted using the emmeans package in R (Lenth, 2020), with the Holm-Bonferroni correction for multiple comparisons.

## 3 Results

Using a categorization task on morphed faces, we measured performance in two experimental sessions. To decipher the contribution of each measure (choices and RT) we further modeled these results using the drift diffusion model (DDM). To assess the impact of attentional stress, we administered each session with three ITI conditions, asking whether increasing the intervals has a larger effect in dyslexia. We first present the measured results, then describe their attribution to separate underlying components by the DDM. In these sections we present the results averaged across ITIs, since there were no significant interactions between session and ITI. The effect of ITI, which was similar in the two groups, is discussed in the final section.

### 3.1 Group differences and the effects of practice

#### 3.1.1 Reduced item-specific learning in IDDs

**Fig. 3:**
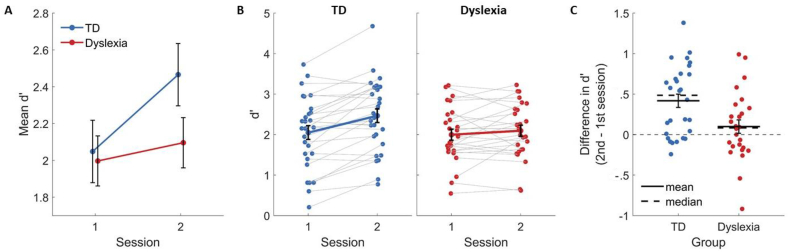
Reduced learning of face categorization in IDDs compared with TDs. (A) The mean categorization d′ of each group in each session. (B) The d′ of each participant in each session. Left – TDs. Right – IDDs. Colored bold lines connect the means. (C) Learning across sessions – the difference between d′ in the second versus the first session at the level of single participants. TDs improved while IDDs did not. Horizontal filled lines denote the mean. Horizontal dashed lines denote the median. In all graphs, values were jittered horizontally for display purposes. Error bars denote the standard error of the mean.

We assessed learning in each group as the change in performance between the first and the second sessions (Fig. 3). To do so, we calculated the sensitivity index, d′. There was a significant main effect of session on d′ [F(1, 52) = 18.14, $\eta_{G}^{2}$ = .019, *p* < .001], and the main group effect was not significant [F(1, 52) = .99, $\eta_{G}^{2}$ = .014, *p* = .323]. Importantly, there was a significant group × session interaction [F(1, 52) = 6.31, $\eta_{G}^{2}$ = .007, *p* = .015]. As shown in Fig. 3A, TDs' d′ significantly increased between sessions [mean ± SD first session = 2.05 ± .9, second session = 2.47 ± .9, F(1, 52) = 23.8, *p* < .001], whereas IDDs' did not [mean ± SD first session = 2 ± .7, second session = 2.1 ± .7, F(1, 52) = 1.47, *p* = .231]. Fig. 3B shows individual participants. It shows that the vast majority of TDs improved their d′ from the first to the second session. In contrast, only about half of IDDs improved in the task, and their improvement was mostly smaller than that of the large group of improved TDs. The d′ of two IDDs even decreased between the sessions by more than .5 (Fig. 3C). Importantly, a significant group by session interaction remains also when these two participants are excluded from calculation (*p* < .05). In general (except these two participants), the d′ difference of IDDs is within the lower limit of TDs' distribution, indicating that IDDs' reduced learning rate is not extreme. Rather, it is within the lower neurotypical quartile. In calculating the sensitivity index d′, we only considered actual responses. However, due to the 1.5 sec response time-out, there was a small percentage of no-response trials, and this percentage was larger in the dyslexia group (see Methods). To make sure that removing these trials from our analysis did not affect the results, we calculated percent correct considering no-response trials as error, and found that the group by session interaction was significant (*p* < .05) also when using this measure.

**Fig. 4:**
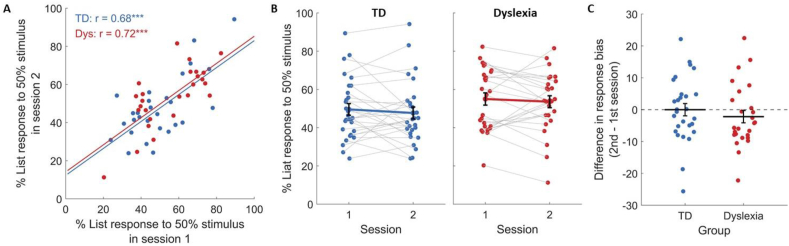
Bias of responses to the 50% stimulus did not change in either group between sessions. (A) The percent of “Liat” responses to the ambiguous 50% stimulus in the second versus first session of each participant. Regression lines are presented for TDs (in blue) and IDDs (in red) separately. r denotes the Pearson correlation. ∗∗∗ denotes *p* < .001 in a two-tailed test. (B) The percent “Liat” responses to the ambiguous 50% stimulus in each session at the level of single participants. Colored bold lines connect the means. (C) The differences between the bias of responses to the ambiguous 50% face in the second versus the first session. The response bias of each participant was defined as the absolute difference between their mean percent of “Liat” responses and chance level (50%). Positive values denote an increase in bias from the first to the second session. Across groups, there was no change in response bias between sessions, suggesting that the learning effect, quantified by d′, was not due to reduced noise (enhanced response consistency for a given stimulus). Horizontal lines denote the mean. In all graphs, values were jittered horizontally for display purposes. Error bars denote the standard error of the mean.

We further asked whether TDs' larger improvement in categorization stems from enhancing their face-specific representation, or from reducing internal noise and consequently increasing decision consistency (the percent of the same responses to a given morph, Gold et al., 1999). To ask that we assessed the bias in responses to the ambiguous 50% stimulus (Fig. 4). Given that there is no correct answer, we could assess how consistent response were (in this case, how biased they were) in each session where it is not confounded with correctness. As shown in Fig. 4A, in both groups there was a broad distribution of single participant responses, though the group average was ∼50%. Assessing the correlation between individuals' responses in the first and second session we found a high consistency in their choices (TDs: r Pearson = .68, *p* < .001; IDDs: r Pearson = .72, *p* < .001; Fig. 4B). To allow comparison between the groups' biases, we calculated for each participant the (absolute) distance of their responses to the ambiguous stimulus from chance [abs(percent of “Liat” responses − 50)] in each session. In an ANOVA on this measure there was no main effect of group [F(1, 52) = .182, $\eta_{G}^{2}$ = .003, *p* = .671] or session [F(1, 52) = .675, $\eta_{G}^{2}$ = .004, *p* = .415], and the group × session interaction was also not significant [F(1, 52) = .651, $\eta_{G}^{2}$ = .004, *p* = .424; Fig. 4B and C]. Namely, participants in both groups were similarly biased in their responses to the 50% stimulus, and their bias did not change from the first to the second session. Neither group showed a reduction in their internal noise, in line with previous studies of face learning (Gold et al., 1999).

Taken together, our results suggest that TDs' learning to better categorize than IDDs is based on enhancing the representation of these faces rather than reducing their internal noise.

#### 3.1.2 RT was longer in dyslexia, yet it similarly decreased with practice in both groups

**Fig. 5:**
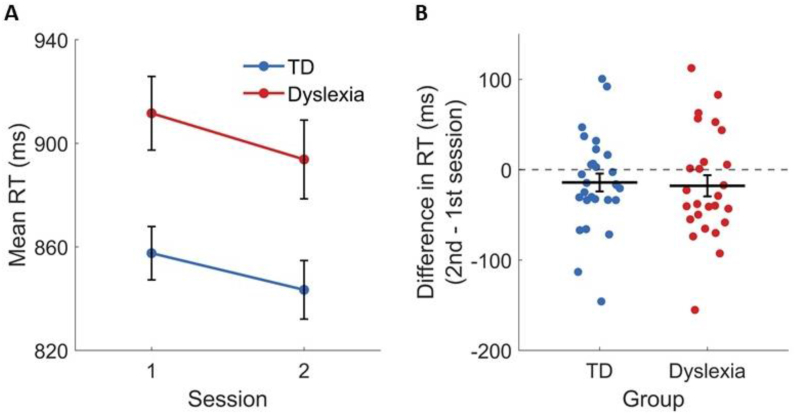
RT was longer in dyslexia throughout the experiment, and decreased similarly in the second session in the two groups. (A) The mean RT of TDs (blue) and IDDs (red) in each session. (B) The difference between RT in the second versus the first session, at the level of single participants. The decrease in RT was similar in the two groups. Horizontal lines denote the mean. Error bars denote the standard error of the mean.

To assess whether response times were longer in dyslexia, and whether they were modified to a different extent than those of TDs, we administered an ANOVA using RT as the dependent variable. There was a main effect of group [F(1, 52) = 10.27, $\eta_{G}^{2}$ = .11, *p* = .002], indicating that TDs' RT (mean ± SD = 850 ± 58 msec) was shorter than IDDs' (mean ± SD = 902 ± 75 msec; Fig. 5A). There was also a significant main effect of session on RT [F(1, 52) = 4.53, $\eta_{G}^{2}$ = .012, *p* = .038], indicating a reduction in RT. But the group × session interaction was not significant [F(1, 52) = .07, $\eta_{G}^{2}$ < .001, *p* = .793]. Namely, as shown in Fig. 5B, in both groups, participants' RT was shorter in the second session (mean ± SD, TDs' first session = 858 ± 67 msec, and second session = 843 ± 68 msec; IDDs' first session = 912 ± 85 msec, and second session = 894 ± 84 msec). However, this difference did not differ between groups.

### 3.2 Drift diffusion modeling (DDM) of the learning effects

To further assess which processing stage is differently affected in TDs and IDDs, we used the DDM, a model that decomposes choices and RT into different constructs of the decision making process (for further details on the model, see Methods). First averaging across ITIs, we asked: 1) Between sessions, whether TDs' larger improvement resulted from a larger increase in the drift rate parameter, reflecting accumulation of sensory information, or in the threshold separation parameter, reflecting a higher criterion for decision making, perhaps due to a change in the strategy of decision making from the first to the second session. 2) Across sessions, whether the longer RT in dyslexia was due to an overall slower accumulation of information (smaller drift rate), or to a longer non-decision time, which is related to non-decisional processes (including non-sensory, motor processes). A third option is a larger threshold separation, meaning that across sessions, IDDs aimed to acquire more evidence before making a decision.

#### 3.2.1 Improved learning in TDs reflects an increase in the drift rate (v)

**Fig. 6:**
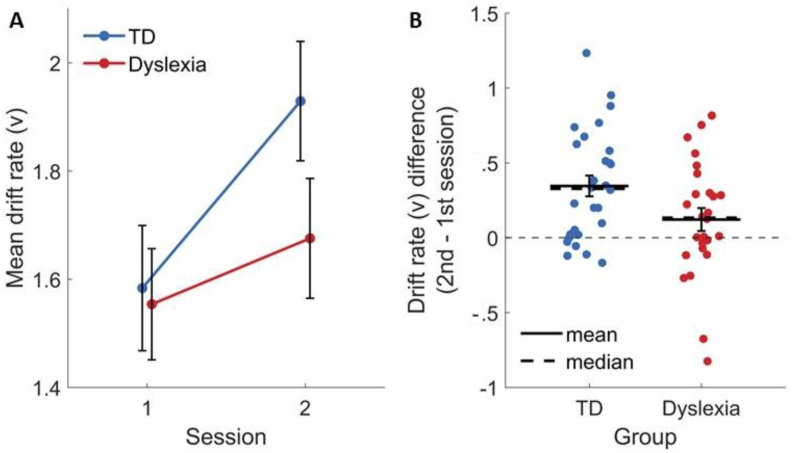
Greater training-induced improvement in face categorization in TDs was modeled in the DDM as a larger change in the drift rate (v) from the first to the second session. (A) The mean estimates of the drift rate (v) by session across ITIs for TDs (blue) and IDDs (red). (B) The difference between the drift rate estimates in the second versus the first session, at the level of single participants. Horizontal filled lines denote the mean. Dashed lines denote the median. Error bars denote the standard error of the mean.

We examined whether the drift rate differed between groups and how was it affected by practice. There was no main effect of group [F(1, 52) = .89, $\eta_{G}^{2}$ = .011, *p* = .351], and indeed, the drift rate in the first session was very similar in the two groups (TDs: mean ± SD = 1.58 ± .91; IDDs: mean ± SD = 1.55 ± .85; Fig. 6A). Thus, despite the longer RT in IDDs (which can be associated with lower drift rate values), our modeling did not find a general difference in the rate of accumulation of face information. However, there was a significant main effect of session [F(1, 52) = 20.7, $\eta_{G}^{2}$ = .03, *p* < .001], indicating cross-session learning. Importantly, there was also a significant group × session interaction [F(1, 52) = 4.75, $\eta_{G}^{2}$ = .007, *p* = .034]. Namely, as shown in Fig. 6B, there was a larger increase in the drift rate of TDs from the first to the second session, compared with IDDs'. Post-hoc tests revealed a significant increase in the drift rate of TDs from the first to the second session [F(1, 52) = 23.51, *p* < .001], where in IDDs, this tendency did not reach significance [F(1, 52) = 2.71, *p* = .11]. Thus, the group learning effect is modeled as a larger increase in the rate of information accumulation in TDs, as a function of task and stimulus experience.

#### 3.2.2 Model parameters that are not associated with learning

##### 3.2.2.1 Longer RT in dyslexia – longer non-decision time (t0)

As discussed above, the drift rate did not differ between groups in the first session. Thus, a good candidate for explaining IDDs' longer RT across sessions was the non-decision time, which models the duration of processes which are not part of the decision process (i.e., initial encoding and response execution). An initial model allowed the non-decision time to change with session, but neither the session main effect nor the group × session and group × session × ITI interactions were significant (in all tests, *p* > .348), and a model comparison test preferred the model where the non-decision time (and threshold separation, see below) did not change with session (see Methods: the mean BIC of the simpler model, 289.65, was better than that of the more complex model, 294.52). Therefore, the non-decision time was allowed to change only with ITI (see the effect of ITI below). There was a main effect of group on the non-decision time [F(1, 52) = 4.12, $\eta_{G}^{2}$ = .057, *p* = .047], reflecting a larger non-decision time in IDDs (mean ± SD = 632 ± 107) compared with TDs (mean ± SD = 588 ± 71; Fig. 9C). Moreover, the model's estimated group difference in non-decision time, ∼50 msec, matches the size of the group difference in RT (Fig. 5). Namely, IDDs' slower RTs are fully explained as reflecting longer non-decisional processes.

##### 3.2.2.2 Inter-trial variability of non-decision time (st0) is longer in dyslexia

The inter-trial variability of the non-decision time was also a free parameter in our modeling (modeled as a single parameter for each participant). It was significantly larger in IDDs (mean ± SD = 446 ± 202) compared with TDs [mean ± SD = 328 ± 137; F(1, 52) = 6.37, $\eta_{G}^{2}$ = .109, *p* = .015].

##### 3.2.2.3 Threshold separation (a) does not differ between groups

We also modeled the threshold separation, which represents the amount of information that participants consider before reaching a decision. A larger threshold separation is associated with more accurate, yet slower decisions. As with the non-decision time, the main effect of session and the group × session and group × session × ITI interactions were not significant in an initial model that allowed the threshold separation to change with session (in all tests, *p* > .22), and a model comparison test preferred a model where the threshold separation (and non-decision time) changes only with ITI (see Methods). The threshold separation did not differ between the groups [F(1, 52) = .27, $\eta_{G}^{2}$ = .004, *p* = .609].

### 3.3 The effects of ITI – behavioral measures and modeling

#### 3.3.1 Behavior – increasing ITIs increased accuracy and response times similarly in both groups

**Fig. 7:**
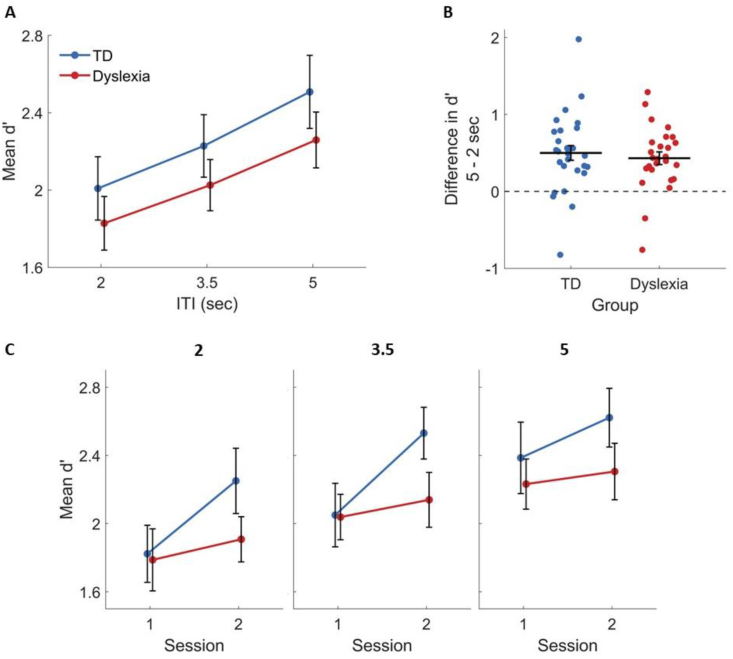
Increasing ITI improved categorization to a similar extent in both groups (A–B), with a tendency for a larger cross-session improvement of TDs in each ITI (C). (A) The mean d′ in each ITI for TDs (blue) and IDDs (red). (B) The difference between d′ in the 5 sec versus the 2 sec ITIs, at the level of single participants. Positive values indicate improved categorization from the shortest to the longest ITI. Horizontal lines denote the mean. (C) The mean d′ in each session for TDs (blue) and IDDs (red) in the three ITI conditions: 2 sec (left), 3.5 sec (middle) and 5 sec (right) shows similar interactions in the three ITIs. Error bars denote the standard error of the mean.

In the second part of the analysis, we asked how changing the ITI affects task performance. For accuracy we used d′, and found a significant main effect of ITI [F(1.96, 101.66) = 28.52, $\eta_{G}^{2}$ = .042, *p* < .001]. Namely – increasing ITI improved performance, although the response time participants were allowed (time out) did not differ between ITIs. Post-hoc tests revealed that there was a significant increase in d′ with ITI between each pair of ITIs for each of the groups (*p* < .026 in all tests), as illustrated in Fig. 7A. Yet, the group × ITI interaction was not significant [F(1.96, 101.66) = .08, $\eta_{G}^{2}$ < .001, *p* = .919], indicating that this effect did not differ between groups (Fig. 7A and B). The group × ITI × session interaction was not significant [F(1.97, 102.62) = .35, $\eta_{G}^{2}$ = .001, *p* = .705]. As shown in Fig. 7C, the group effect of learning was similar in the three ITIs.

**Fig. 8:**
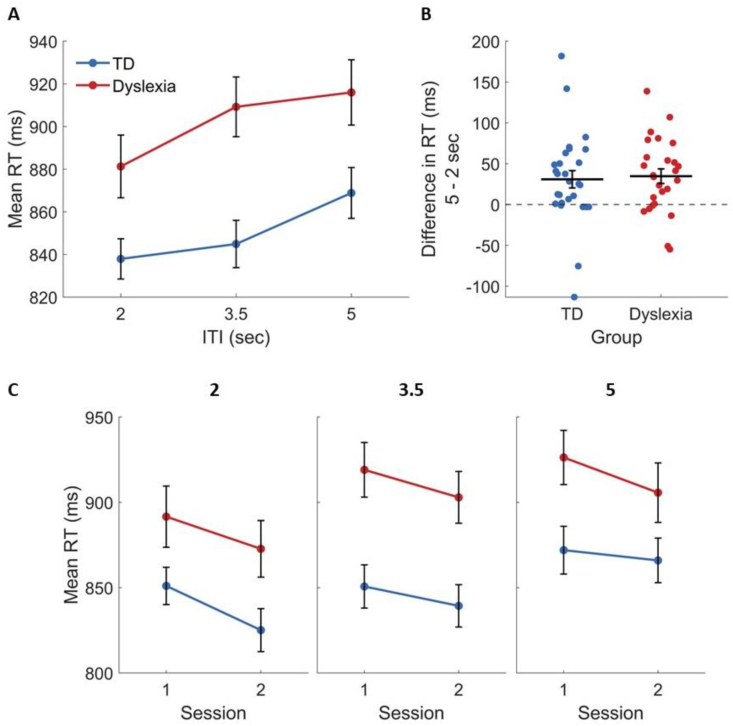
RT increased with ITI in both groups, and similarly decreased across sessions in each ITI. (A) The mean RT increases with longer ITIs in both TDs (blue) and IDDs (red). (B) The difference in RT in the 5 sec versus 2 sec ITIs, at the level of single participants. Horizontal lines denote the mean. (C) The mean RT of TDs (blue) and IDDs (red) in each session, in each ITI: 2 sec (left), 3.5 sec (middle) and 5 sec (right). The group × session × ITI effect was not significant [F(1.71, 88.82) = .5, $\eta_{G}^{2}$ = .001, *p* = .577], i.e., the effect of ITI did not interact with cross-session learning of the two groups. Error bars denote the standard error of the mean.

Fig. 8 shows the same ITI analysis as Fig. 7, this time with RT as the dependent measure. As in d′, there was a main effect of ITI [F(1.97, 102.46) = 11.78, $\eta_{G}^{2}$ = .03, *p* < .001], reflecting an increase in RT with increased ITI (Fig. 8A and B). As shown in Fig. 8A, the main increase in TDs was between 3.5 and 5 sec (*p* = .016) and the main increase in IDDs was between 2 and 3.5 sec (*p* = .011). However, here too, the group × ITI interaction was not significant [F(1.97, 102.46) = 1.58, $\eta_{G}^{2}$ = .004, *p* = .211], indicating that the effect was similar in the two groups, and both showed a significant increase between 2 and 5 sec in post-hoc tests (*p* < .007 in both groups; Fig. 8B). In summary, although the duration of the stimulus (which was also the duration allowed for response) was fixed throughout the experiment, increasing the time interval between trials increased both accuracy and RT. However, it did so similarly for the two groups, and without affecting the learning dynamics.

#### 3.3.2 DDM – larger ITIs increase drift rate (v) and non-decision time (t0)

##### 3.3.2.1 Increasing ITI increased drift rate (v)

**Fig. 9:**
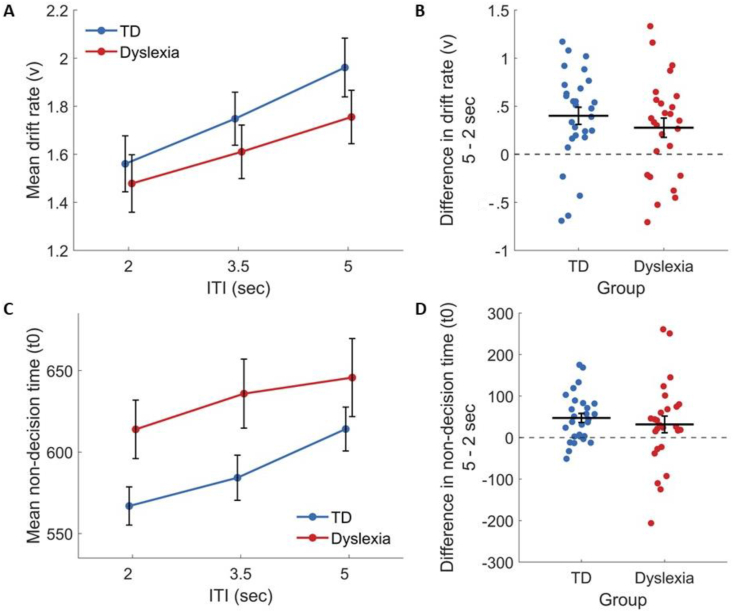
The behavioral effects of increasing the ITI are explained as an increase in both the drift rate (v) and the non-decision time (t0) in both groups (a). Left – the mean parameter estimates in each ITI for TDs (blue) and IDDs (red). Right – The difference in parameter estimates in the 5 sec versus the 2 sec ITIs, at the level of single participants. Horizontal lines denote the mean. (A) and (B) the drift rate (v); (C) and (D) the non-decision time (t0). Error bars denote the standard error of the mean.

Across groups, increased ITI was associated with both an improvement in d′ and longer RTs. Asking whether this increase was associated with increased efficiency in accumulating stimulus information, we modeled this effect using the DDM. An ANOVA on the estimated drift rates revealed a main effect of ITI [F(1.89, 98) = 15, $\eta_{G}^{2}$ = .041, *p* < .001], namely – increased drift rates with longer ITIs (Fig. 9A and B). This effect did not differ significantly between the groups [the group × ITI interaction was not significant; F(1.89, 98) = .5, $\eta_{G}^{2}$ = .001, *p* = .594]. The ITI × group × session interaction was also not significant [F(1.99, 103.47) = 1.47, $\eta_{G}^{2}$ = .003, *p* = .235]. Thus, the finding of better accuracy as ITI was increased is explained in both groups by an improved information accumulation process when the experiment pace is slower.

##### 3.3.2.2 Increasing ITI increased non-decision time (t0)

The increase in drift rate cannot explain the effect of ITI on RT, since larger drift rates are associated on average with faster responses, while we found slower responses in larger ITIs. Thus, we tested the effect of ITI on the non-decision time. There was a main effect of ITI [F(1.91, 99.26) = 7.29, $\eta_{G}^{2}$ = .032, *p* = .001; Fig. 9C and D]. Namely, increasing the time between trials was associated with longer non-decisional processes measured during the trials. There was no significant interaction between group and ITI [F(1.91, 99.26) = .52, $\eta_{G}^{2}$ = .002, *p* = .591], indicating that this effect did not differ between groups.

##### 3.3.2.3 No change in threshold separation (a)

Finally, when testing the threshold separation, we found no effect of ITI [F(1.78, 92.74) = 1.64, $\eta_{G}^{2}$ = .008, *p* = .203], and the group × ITI interaction was not significant [F(1.78, 92.74) = .38, $\eta_{G}^{2}$ = .002, *p* = .659]. Thus, the amount of information needed in order to make a decision was not affected by ITI, in either group.

Overall, we found that in blocks in which ITI was longer, namely – the trial rate was slower, participants were both more efficient in their information accumulation, and slower in their non-decisional processes. Yet, these effects were similar in TDs and IDDs, and were not affected by practice. The similarity of increased performance with increased ITI in both groups suggests that “sluggish attention”, leading to the requirement of longer inter-stimulus intervals for similar performance levels in IDDs (Hari & Renvall, 2001) does not account for reduced learning in dyslexia.

## 4 Discussion

In face categorization of morphs of two unfamiliar faces, IDDs' accuracy was initially similar to TDs', but TDs improved more with training. Modeling behavior with the DDM revealed that this increase is fully attributed to a larger increase in the rate of information accumulation in TDs. RT was longer in dyslexia throughout the experiment with no group difference in learning-related dynamics, which was explained by the DDM as reflecting longer non-decisional processes. Finally, increasing inter-trial intervals (2–5 sec) led to both better accuracy and longer RTs, with similar effects in both groups, which were modeled by larger drift rates and longer non-decision times. Taken together, we found that given the same amount of exposure to specific, similar faces, IDDs' acquisition of expertise in categorizing these faces was reduced compared with TDs, in spite of careful matching of participants for non-verbal cognitive abilities (Table 1).

### 4.1 A domain-general shallower learning slope in dyslexia

In the current study, we used faces as an example for a type of stimulus which is not directly related to reading or phonology. Our results imply that given equal exposure, IDDs' item-specific learning is reduced across modalities and stimuli types. This is in line with previous reports for speech stimuli (reviewed in Banai & Ahissar, 2018), and has been expanded here to another modality, suggesting a domain-general property.

A shallower learning slope as a function of exposure to stimuli in dyslexia has been recently hypothesized (Kimel et al., 2020). Since learning does not saturate (Crossman, 1959; Heathcote et al., 2000), this hypothesis entails that while both groups improve with exposure, group differences between TDs and IDDs will increase. This reasoning was used by Kimel et al. (2020) to explain the robust findings of IDDs’ poorer scores in span tasks, which typically use very frequent speech items such as digits. They tested how long-term exposure to the stimuli affects performance in such tasks by comparing the spans of adult individuals with and without dyslexia for frequent and infrequent syllables, and found that TDs benefitted from syllabic long-term frequency more than IDDs, suggesting better long-term learning of these categories. A similar effect of syllable frequency was also observed using the Hebb-learning paradigm (Kimel et al., 2022).

### 4.2 Neural underpinnings

Continuing a line of work on learning of sound categories in dyslexia, where reduced learning in dyslexia was related to reduced neural adaptation in auditory areas (Gertsovski & Ahissar, 2022), we now observed behavioral difficulties with learning of faces. This is consistent with reports of diminished adaptation to faces in IDDs' face processing areas (Perrachione et al., 2016), which were attributed to less efficient integration between bottom-up sensory processing and top-down predictions (Beach et al., 2022). Faster decay of adaptation in dyslexia is expected to yield a noisier perceptual memory trace; Consequently, the next exposure to the stimulus is less effective, and results in less refined representations of the categories in long-term memory.

This idea is related to our findings associating slower learning in IDDs with a smaller increase in the drift rate parameter of the DDM. A more complex (more bits of information) representation of categories (in this case, face categories) in TDs following the same amount of exposure allows them to retrieve more informative features of a new stimulus, which increases their rate of information accumulation. When a representation has more bits of information, each bit can be more informative, potentially increasing the effective rate of information retrieval with the same actual processing rate. This interpretation is also in line with Lieder et al. (2019), who calculated IDDs' learning of stimuli distribution based on patterns of bias in judgments of pitch in a serial discrimination task, and found that it was cruder than that of TDs'.

IDDs' reduced learning of faces suggests that at least in this population there is no trade-off between reading and face identification. This seems in contrast to the neuronal recycling hypothesis, which, in its strong version, would predict such a trade-off (Dehaene & Cohen, 2007), since the acquisition of cultural skills such as reading is expected to yield repurposing of brain areas that were previously dedicated to processing faces, perhaps in the left hemisphere, that will be gradually used for reading. However, a recent fMRI study that tested the neuronal recycling hypothesis by scanning children before and after reading acquisition, suggested that areas selective to word processing were previously not selective to faces, and in general were only weakly specialized (Dehaene-Lambertz et al., 2018).

### 4.3 Implicit category learning in dyslexia

The literature of category learning traditionally distinguishes between rule-based learning, based on explicit reasoning, and information-integration learning, which involves more implicit distinctions that are difficult to describe verbally (Ashby & Maddox, 2005). Our task, in which two unfamiliar faces were morphed, and which included feedback only during an initial introduction phase on the original (100%) faces, involves several complex dimensions on which categorization could have been based. Thus, it requires implicit learning.

IDDs' difficulties in our study are consistent with previous studies, which found difficulties in implicit learning of non-linguistic categories in dyslexia. For example, implicit (but not explicit) learning of categories based on a combination of shape and color was poorer in students with lower reading abilities. In this case, the groups' cognitive abilities were not matched, so the difference might not be specifically associated with dyslexia (Sperling et al., 2004). A shallower learning slope in dyslexia was also found in a probabilistic category learning task (the weather prediction task; Gabay et al., 2015). However, the challenge in this task was mainly memory rather than perceptual, i.e., learning the complex association between clearly distinct shapes and their implied probabilities. In another study by the same group, incidental perceptual learning of auditory categories was poorer in dyslexia both for unidimensional and multidimensional categories (Gabay & Holt, 2015). Importantly, our study extends these findings to a different modality, with very different stimuli, for which expertise is associated with the right hemisphere.

### 4.4 Slower categorical learning is a core deficit

Our observation of poor categorical learning for faces has a unique contribution to assessing causality between reading and categorization learning. In speech perception, causality probably operates in both directions, which are difficult to disentangle: sensitivity to non-native speech contrasts was shown to decrease with early school experience, presumably due to an increase in reading-related activities in these years, which entail ignoring non-native variations of phonemes when mapping them onto written graphemes (Horlyck et al., 2012). Thus, IDDs' perception of speech categories may be less categorical because they have less experience with reading. But this does not apply in the current study, which used faces as stimuli. Hence, our observation of slower categorical learning of new faces among IDDs, suggests that slower categorical learning of speech stimuli reflects a core deficit of IDDs, rather than merely reflecting their reduced reading experience. Beyond the issue of causality, by “core deficit” we mean first, that slower item-specific learning consistently characterizes the dyslexia group. In our results, it characterizes the majority of IDDs, though it doesn't segment the dyslexia group to two separate modes (subgroups). Namely, ∼half of the IDDs (versus most of the ITDs) showed some cross-session improvement (Fig. 3), yet this improvement was smaller than that of most TDs. Second, reduced learning of specific faces or speech stimuli can be explained as a manifestation of a core cross-modal reduction in the rate of item-specific categorical learning.

### 4.5 Long-term face expertise in dyslexia

The domain-general account discussed here raises several questions. How should a slower learning rate affect IDDs' long-term expertise in face processing? And what does it mean for a disorder that is defined specifically based on reading difficulties? Generally, we expect that with additional exposure, group differences between TDs and IDDs will increase (Kimel et al., 2020). In the current experiment, we used morphs of two unfamiliar faces. Whether there should be a group difference right from the start, due to long-term expertise with other faces, is beyond the scope of our prediction. Perceptual learning is attained through task and stimulus-specific processes (Ahissar & Hochstein, 2004), and human expertise for face identity may be very specific to the faces that individuals were exposed to (Young & Burton, 2018). Thus, a shallower learning slope may result in substantial group differences in categorizing familiar faces, but smaller differences (or none at all) for unfamiliar faces. This is in line with our inconclusive findings regarding group differences at the first stages of the experiment. When we tested performance (d′), no group differences were found in the first session. However, in more peripheral measures of performance, there were differences already from the beginning: lower RT in dyslexia throughout the experiment, IDDs needing marginally more introduction sessions to pass our predefined threshold of basic learning of the two endpoint faces, and more no-response trials in the dyslexia group. This suggests a somewhat lower face categorization performance in dyslexia overall, which is more strongly evident when testing learning of specific items.

If indeed the acquisition of face categories is slower in dyslexia, we would expect that phenomena which characterize face expertise would be reduced. Though such effects were reported in previous studies (Sigurdardottir et al., 2018; Smith-Spark & Moore, 2009), a well-known phenomenon, the other-race effect, i.e., people are worse at discriminating between faces belonging to other races compared to own-race faces, was found adequate (Sigurdardottir et al., 2019). This observation seems inconsistent with our prediction. However, following the discussion above, this is not a stimulus-specific phenomenon. Broad phenomena such as the other-race effect may be intact in dyslexia, developed through the vast exposure to faces throughout life, even if item-specific learning of face identity (which is the type of learning assessed in our study) is slower.

As for the meaning of a general account for a disorder with a specific diagnosis, there are several possible explanations. One option is that among the different skills affected, some (for example, phonological processing) are affected more than others. Another possibility is that different difficulties have different practical implications, which affect the accessibility of their assessment and diagnosis. Reading is an academic skill, and as such reading skills are more commonly assessed than face recognition skills. This is demonstrated by the fact that research on congenital prosopagnosia began only relatively recently, in the past 20–30 years (Behrmann & Avidan, 2005). It may be that many IDDs have difficulties in categorical learning of faces, but these are not diagnosed. Importantly, as evident in our results, learning face identification among our participants is mildly reduced, and is mostly in the lower range of TDs' learning abilities.

### 4.6 DDM in dyslexia

The current study is the first to use the DDM to model IDDs' performance in a face categorization task. However, the DDM was used in two previous studies of perceptual discriminations in dyslexia. Both studied global-motion discrimination among children with dyslexia (Manning et al., 2022; O'Brien & Yeatman, 2021). Both studies found reduced drift rates in dyslexia, implying that the rate of evidence accumulation of motion information is slower. In the current study, we did not find an overall smaller drift rate in dyslexia, only a smaller change in the parameter between sessions. It may be that IDDs have a specific difficulty with extracting motion information, that is not evident in our static face processing task. Results related to the non-decision time in dyslexia were not consistent between the two mentioned studies; O'Brien & Yeatman found that poorer readers have longer and more variable non-decision times, in line with our observation that IDDs had longer RTs, which was attributed by the DDM to longer non-decision time. However, this effect was not replicated in Manning et al. We should note that in general, the observation of longer RTs in dyslexia is consistent with previous studies of face processing in dyslexia. Not all of these studies reported RT results, but of those that did, several reported slower responses in IDDs (Gabay et al., 2017; Tarkiainen et al., 2003; Åsberg Johnels et al., 2022).

### 4.7 ITI and attentional effects

Finally, an important observation in the current study is the lack of interaction between group and ITI. We found that increasing the ITI increased both d′ and RT, modeled as an increase in both drift rate and non-decision time. It is thus interesting to note that to some extent, difficulties in categorization can be overcome by relaxing the task pace. However, these effects did not differ between TDs and IDDs. Sensory and motor deficits in dyslexia were previously attributed to ‘sluggish attentional shifting’ (Hari & Renvall, 2001), a cross-modal difficulty in disengaging attention and attending on an incoming input. According to this theory, group differences in different paradigms may relate to temporal parameters such as the ITI, with relatively short ITIs being too short for IDDs to be fully attentive to the stimuli. Since there was no group difference in the effect of elongating ITI on performance in our study, we conclude that reduced learning in IDDs does not stem from temporally-sluggish attention.

In addition, a possible explanation for the reduced improvement in categorization of IDDs in the second session could be greater fatigue in this group, perhaps due to group differences in sustained attention. While we cannot unequivocally rule out this explanation, we think that it is less likely. The two sessions were separated by a mandatory ∼20 min break, during which the participants could rest. This should have helped them avoid an effect of fatigue. In addition, while d′ did not increase in dyslexia in the second session, RT did not show a sign of a differential effect of fatigue, since it similarly decreased in the two groups from the first to the second session.

In summary, direct behavioral measures and modeling the rate of accumulation of sensory information, showed significantly better perceptual learning of face categorization in TDs compared with IDDs. This effect, shown for a stimulus which is not related to reading difficulties, and for which exposure does not depend on reading abilities, suggests a core domain-general category learning deficit in dyslexia.

## Data statement

The conditions of our ethical approval for this study do not permit uploading of data to public data repositories. Readers seeking access to the data should contact the corresponding author. Access will be granted in accordance with ethical procedures governing the reuse of sensitive data. The scripts used for data analysis are available at: https://osf.io/tvq9c. No part of the study procedures or analysis plans was preregistered prior to the research being conducted. We report how we determined the sample size, all data exclusions (if any), all inclusion/exclusion criteria, whether inclusion/exclusion criteria were established prior to data analysis, all manipulations, and all measures in the current study.

## Open practices

The study in this article earned Open Material Badge for transparent practices. The material used in this study are available at https://osf.io/tvq9c.

## CRediT authorship contribution statement

**Ayelet Gertsovski:** Writing – review & editing, Writing – original draft, Software, Methodology, Investigation, Formal analysis, Conceptualization. **Odeya Guri:** Software, Methodology, Investigation, Conceptualization. **Merav Ahissar:** Writing – review & editing, Writing – original draft, Supervision, Methodology, Funding acquisition, Conceptualization.
